# The nature of psychopathology

**DOI:** 10.1002/jcv2.12122

**Published:** 2022-11-26

**Authors:** Henrik Larsson, Erik Pettersson

**Affiliations:** ^1^ School of Medical Sciences Örebro University Örebro Sweden; ^2^ Department of Medical Epidemiology and Biostatistics Karolinska Institutet Stockholm Sweden

**Keywords:** categories, dimensions, genomics, *p*‐factor, psychopathology

## Abstract

Readers of JCPP Advances known that the dominant classification system (Diagnostic and Statistical Manual of Mental Disorders) in psychiatry and psychology conceptualizes psychopathology as discrete diagnostic categories. This measurement model builds on a strong assumption of a clear discontinuity between individuals who meet criteria for a diagnosis and those who don't fulfill diagnostic criteria. During the past decades we have seen huge efforts to test this assumption and explore alternative models, such as research from the hierarchical taxonomy of psychopathology consortia. Key findings of these efforts are reviewed and discussed in the December issue of JCPP Advances.

JCPP Advances aims to lead and influence the field of child psychology and psychiatry. We are committed to stimulate discussion around fundamental questions via editorials, commentaries, and reviews. The review papers from Lahey et al. (2022) and Plomin (2022) and the associated commentaries by Hinshaw (2022) and Sonuga‐Barke (2022) in the December issue of JCPP Advances represent excellent examples of when such discussions are at the highest level.

Readers of JCPP Advances know that the dominant classification system (Diagnostic and Statistical Manual of Mental Disorders [DSM]) in psychiatry and psychology conceptualizes psychopathology as discrete diagnostic categories. This measurement model builds on a strong assumption of a clear discontinuity between individuals who meet criteria for a diagnosis and those who don't fulfill diagnostic criteria. During the past decades we have seen huge efforts to test this assumption and explore alternative models, such as research from the hierarchical taxonomy of psychopathology consortia (Kotov et al., 2018). Key findings from such research are reviewed and discussed in the above mentioned four publications in JCPP Advances.

## DIMENSIONAL VERSUS CATEGORICAL MODELS OF PSYCHOPATHOLOGY

In the brave review from Lahey et al. (2022), readers are directed to findings indicating that the measurement of psychopathology as dimensions is more reliable and valid than the categorical model of psychopathology (i.e., DSM). Lahey et al. (2022) also review previous research indicating that the dimensions better address *heterogeneity* (e.g., individuals with the same diagnosis vary considerably in their symptom profiles), *comorbidity* (i.e., individuals may fulfill criteria for two of more diagnosis) and *developmental change* (i.e., individuals may change their diagnostic status across time) than the categorical model based on DSM. Finally, Lahey et al. (2022) discuss findings from a large number of taxometric studies highlighting that categories or taxa rarely are found to underlie measures of psychopathology.

The impressive review paper by Plomin (2022), that outline the past, present and future directions of behavioral genomic research, also address the dimensional versus categorical debate. Plomin (2022) presents two major behavioral genomic findings that support the notion that the nature of psychopathology is dimensional rather than categorical. First, twin and molecular genetic studies report that diagnosed conditions (e.g., attention deficit hyperactivity disorder [ADHD]) yield substantial genetic correlations with corresponding quantitative traits (e.g., ADHD symptoms). Second, polygenic scores derived from a case‐control genome‐wide association analysis of diagnosed disorders (e.g., ADHD) predicts the corresponding quantitative traits (e.g., ADHD symptoms) in community samples.

Taken together, a substantial body of research suggests that psychopathology is likely dimensional phenotypes rather than categorical entities. One reasonable hypothesis is, therefore, that a dimensional approach may provide a more effective approach to identify the causes and etiology of child and adolescent psychopathology compared to the approach based on diagnostic categories. It makes sense scientifically to adopt a dimensional view in research of psychopathology, but are we ready to fully replace the available diagnostic nosology with a dimensional model?

The commentaries from Hinshaw (2022) and Sonuga‐Barke (2022) highlight that the societal implications and clinical utility of replacing the available categorical model with dimensions remain unclear. Their insightful discussion covers two important themes that needs careful deliberation, discussion and further research. The first theme covers questions related to stigma. For example: Does diagnosis necessarily always increase stigma? What terminology should be used to minimize stigma related to psychopathology? The second theme deal with problems related to implementation of a substantially different diagnostic system for psychopathology. For example: Can clinical decision making about psychopathology ever be anything other than categorical? What cost would be associated with replacing the categorical system with a dimensional approach? Will replacing the available diagnostic nosology with a dimensional model improve the care provided to people in the clinic? How do we keep up a strong link between science and clinical practice to facilitate translational impact? As noted by others (Haeffel et al., 2022; Zimmerman, 2021), more high‐quality studies and reviews are needed before we are ready to replace the available diagnostic nosology with a dimensional model.

## THE UNDERLYING STRUCTURE OF PSYCHOPATHOLOGY

The review papers from Lahey et al. (2022) and Plomin (2022) also presents findings that challenge other important features of the categorical DSM classification system, in particular it's focus on symptom presentation—rather than the underlying etiology—and the assumption that different diagnosis are distinct.

First, research have convincingly demonstrated that almost all forms of psychopathology are positively correlated with high rates of comorbidity. This observation challenges the assumption that different diagnoses are distinct and indicate that they may have common underlying causes. Research at the phenotypic symptom/trait level suggest that the correlated nature of psychopathology can be adequately captured by a hierarchical model that include a general factor of psychopathology (*p* factor) at the top and several specific factors at lower levels such as neurodevelopmental, externalizing and internalizing problems (Kotov et al., 2018; Lahey et al., 2021). The so called “*p* factor” model of psychopathology suggests that different forms of psychopathology are unified by a general factor—analogous to the general factor (*g* factor) of cognitive abilities that accounts for the positive correlations among scores on the subtests of intelligence (Caspi & Moffitt, 2018; Pettersson et al., 2020). Thus, there may be a single underlying factor that accounts for individuals' propensity to develop any and every form of psychopathology. These findings indicate that there might be alternative models that better align the nosology with data on the actual structure of psychopathology.

Second, a substantial number of behavioral genomic studies indicate that comorbidity is mostly genetic in origin and that genetic overlap among different conditions might reflect, at least partly, a transdiagnostic, genetically influenced general factor of psychopathology. Plomin (2022) predicts that future behavioral genomic research will move beyond documenting the inadequacies of current nosology to reveal the genetic architecture of psychopathology. This ambitious goal requires that future behavioral genomic research remove the circularity (i.e., it has necessarily relied on the available diagnostic system) that has constrained the available genomic research of psychopathology. An important next step is to enrich the available genome‐wide, case‐control studies with quantitative measures of the underlying building blocks for the corresponding diagnosis. A more important, but even more challenging task for future behavioral genomic studies is to build the structure from the ground up (which was the case for personality and intelligence many years ago), using batteries of psychopathology assessed as dimensions in samples of unrelated individuals.

## CONCLUSION

The dominant classification system in psychiatry and psychology conceptualizes psychopathology as discrete diagnostic categories. A substantial number of studies have asked fundamental questions about the nature of psychopathology related to dimensionality and the underlying structure. Findings from these studies challenge critical assumptions of this categorical model. Further developments of a theory‐based classification system that better align the nosology with data on the actual structure and etiology of psychopathology is, therefore, an important scientific goal.

## AUTHOR CONTRIBUTIONS

**Henrik Larsson**: Writing – original draft; Writing – review & editing. **Erik Pettersson**: Writing – original draft; Writing – review & editing.

## CONFLICT OF INTEREST

Henrik Larsson reports receiving grants from Shire Pharmaceuticals; personal fees from and serving as a speaker for Medice, Shire/Takeda Pharmaceuticals and Evolan Pharma AB; and sponsorship for a conference on attention‐deficit/hyperactivity disorder from Shire/Takeda Pharmaceuticals and Evolan Pharma AB, all outside the submitted work. Henrik Larsson is editor‐in‐chief of JCPP Advances. Erik Pettersson serves on the Editorial Advisory Board for JCPP Advances.

## References

[jcv212122-bib-0001] Caspi, A. , & Moffitt, T. E. (2018). All for one and one for all: Mental disorders in one dimension. American Journal of Psychiatry, 175(9), 831–844. 10.1176/appi.ajp.2018.17121383 29621902 PMC6120790

[jcv212122-bib-0002] Haeffel, G. J. , Jeronimus, B. F. , Kaiser, B. N. , Weaver, L. J. , Soyster, P. D. , Fisher, A. J. , Vargas, I. , Goodson, J. T. , & Lu, W. (2022). Folk classification and factor rotations: Whales, sharks, and the problems with the hierarchical taxonomy of psychopathology (HiTOP). Clinical Psychological Science, 10(2), 259–278. 10.1177/21677026211002500 35425668 PMC9004619

[jcv212122-bib-0009] Hinshaw, S. P. (2022). Psychological problems, biomedical models, and stigma: A commentary on Lahey et al. JCPP Advances, e12119. 10.1002/jcv2.12119 PMC10242839 37431420

[jcv212122-bib-0003] Kotov, R. , Krueger, R. F. , & Watson, D. (2018). A paradigm shift in psychiatric classification: The hierarchical taxonomy of psychopathology (HiTOP). World Psychiatry, 17(1), 24–25. 10.1002/wps.20478 29352543 PMC5775140

[jcv212122-bib-0004] Lahey, B. B. , Moore, T. M. , Kaczkurkin, A. N. , & Zald, D. H. (2021). Hierarchical models of psychopathology: Empirical support, implications, and remaining issues. World Psychiatry, 20(1), 57–63. 10.1002/wps.20824 33432749 PMC7801849

[jcv212122-bib-0005] Lahey, B. B. , Tiemeier, H. , & Krueger, R. F. (2022). Seven reasons why binary diagnostic categories should be replaced with empirically sounder and less stigmatizing dimensions. JCPP Advances, 2(4), e12108. 10.1002/jcv2.12108 PMC10242872 37431412

[jcv212122-bib-0006] Pettersson, E. , Larsson, H. , D'Onofrio, B. M. , Bolte, S. , & Lichtenstein, P. (2020). The general factor of psychopathology: A comparison with the general factor of intelligence with respect to magnitude and predictive validity. World Psychiatry, 19(2), 206–213. 10.1002/wps.20763 32394574 PMC7215062

[jcv212122-bib-0007] Plomin, R. (2022). The next 10 years of behavioural genomic research. JCPP Advances, 2(4), e12112. 10.1002/jcv2.12112 PMC10242940 37431418

[jcv212122-bib-0010] Sonuga‐Barke, E. J. S. (2022). Commentary: “If it don’t fit, don’t force it”: Are complex clinical decisions inevitably categorical, based on their practical purpose and psychological nature? A reflection on the Lahey, Tiemeier & Krueger (2022) manifesto. JCPP Advances, e12120. 10.1002/jcv2.12120 PMC10242901 37431422

[jcv212122-bib-0008] Zimmerman, M. (2021). Why hierarchical dimensional approaches to classification will fail to transform diagnosis in psychiatry. World Psychiatry, 20(1), 70–71. 10.1002/wps.20815 33432768 PMC7801851

